# Psychological Impact of Distance Learning on Children and Adolescents in Saudi Arabia: A Multi-City Analysis of Behavioral and Mental Health Outcomes During the COVID-19 Pandemic

**DOI:** 10.3390/children11121551

**Published:** 2024-12-20

**Authors:** Samah H. Alkhawashki, Maram H. AlTuwairqi, Ahmad H. Almadani, Afnan A. Almarshedi, Rahaf Alasiri, Noha A. Mobeireek, Mishaal R. Alrashoud, Noura A. Abouammoh, Fatimah S. Alshahrani, Areej A. AlFattani, Shuliweeh Alenezi

**Affiliations:** 1 Department of Psychiatry, College of Medicine, King Saud University, Riyadh 11472, Saudi Arabia; 2 SABIC Psychological Health Research and Applications Chair (SPHRAC), Department of Psychiatry, College of Medicine, King Saud University, Riyadh 11451, Saudi Arabia; 3 Department of Psychiatry, Prince Sultan Military Medical City, Riyadh 11159, Saudi Arabia; 4 King Abdullah International Medical Research Center, Jeddah 22384, Saudi Arabia; 5 College of Medicine, King Saudi bin Abdulaziz University for Health Sciences, Jeddah 21498, Saudi Arabia; 6 King Abdullah International Medical Research Center, Riyadh 11481, Saudi Arabia; 7 Department of Psychiatry, Dammam Medical Complex, Dammam 32253, Saudi Arabia; 8 Department of Family and Community Medicine, King Saud University, Riyadh 11451, Saudi Arabia; 9 Department of Medicine, King Saud University, Riyadh 11451, Saudi Arabia; 10 Biostatistics, Epidemiology and Scientific Computing, King Faisal Specialist Hospital and Research Center, Riyadh 11211, Saudi Arabia

**Keywords:** distance learning, virtual schooling, mental health, students, youth, Saudi Arabia

## Abstract

Background: The COVID-19 pandemic triggered a global transition to distance learning, which significantly impacted children’s mental health. In Saudi Arabia, remote education began on 8 March 2020, lasting between 1.5 to 2.5 years. This study aims to explore the psychological effects of distance learning on children and adolescents, with a focus on mental health challenges and coping mechanisms. Methods: A cross-sectional study was conducted using an online survey distributed to parents of children aged 6 to 18 in the major metropolitan areas of Jeddah and Riyadh. The survey included demographic questions and the Arabic version of the Vanderbilt ADHD Diagnostic Rating Scale, a tool for assessing behavioral challenges, anxiety, and symptoms of attention deficit hyperactivity disorder (ADHD). Results: A total of 71.6% of families reported a positive experience with distance learning. A significant correlation was found between parents’ marital status and children’s ability to cope with remote education. Interestingly, children without ADHD symptoms experienced three times more negative outcomes than those with ADHD symptoms. However, despite reporting fewer negative experiences, children with ADHD exhibited increased symptom severity and academic difficulties. Of the students, 5.4% were diagnosed with predominantly inattentive ADHD, 1.8% with predominantly hyperactive/impulsive ADHD, and 3.9% with combined ADHD. Additionally, 7.2% of students screened positive for oppositional defiant disorder, 1.5% for conduct disorder, and 6.6% for anxiety or depression. Children from separated or divorced families were more likely to exhibit ADHD symptoms (*p* = 0.002). Children with ADHD symptoms reported a more positive experience with distance learning (*p* < 0.05). Conclusion: This study represents the first comprehensive, multi-city investigation in Saudi Arabia examining the relationship between distance learning, sociodemographic factors, and mental health symptoms in children. The findings highlight the psychological challenges faced by children during the pandemic and emphasize the need for targeted interventions to support both mental health and academic outcomes. These results offer valuable insights for future research and inform strategies to address children’s well-being in scenarios involving distance learning.

## 1. Introduction

The COVID-19 pandemic caused widespread disruption, significantly impacting the routines and education of children and adolescents worldwide. One of the most notable changes was the shift to distance learning to maintain physical distancing and manage the spread of the virus [1,2]. This transition was mandated in Saudi Arabia for all K–12 schools and higher education institutions starting 8 March 2020. The duration of remote learning varied between 1.5 and 2.5 academic years, depending on factors such as students’ age, vaccination status, and the educational system they were enrolled in, as documented by the Ministry of Education (MOE) in its report on efforts to combat COVID-19 [3].

In the early stages of the pandemic, numerous studies sought to assess the mental health impact of distance learning on students. For example, a survey of 612 adolescents aged 13 to 18 in the United Kingdom reported mental health symptoms in 53.3% of females and 44.0% of males. Anxiety affected 59.6% of males and 47.4% of females, while depressive symptoms were found in 21.9% of males and 19.4% of females [4]. The mental health challenges associated with remote education included anxiety, depression, loneliness, and perceived stress [5,6]. Similarly, in the United States, a study of first-year college students revealed that anxiety prevalence increased from 18.1% to 25.3% and depression from 21.5% to 31.7% within the first four months of the pandemic [7]. In China, a study involving 7143 college students showed a significant correlation between anxiety symptoms and disruptions in daily life and academics [8].

Poor academic performance in the context of distance education was found to be linked with concentration challenges, shorter attention spans, exam-related stress, COVID-19 contagion anxiety, and depressive symptoms [6,9]. Conversely, students with a positive perception of distance learning, family support, and resilience skills were better able to manage educational challenges [6,10].

A survey-based study in Malaysia assessed students’ demographics, academic challenges, and perceptions of remote education while screening for mental health issues using the DASS-21. It reported that 29.4% of university students experienced depression, 51.3% anxiety, and 56.5% stress, with older students showing lower prevalence rates [11]. Another large-scale study using the parent version of the Strengths and Difficulties Questionnaire (SDQ) found that remote learning most negatively affected older children and those from Black, Hispanic, or low socioeconomic backgrounds [12]. Similarly, a survey of Mexican parents of children aged 4 to 15 highlighted behavioral issues, sleep disturbances, and increased screen time as critical challenges of distance education [13]. Interestingly, the perceived threat of COVID-19 among students was correlated with their parents’ perceptions, and parents’ stress levels were positively associated with students’ stress [14].

The topic also gained attention among child age groups. For instance, Panchal U. et al. [15] conducted a systematic review study aiming to review the literature on the effects of the lockdown during the COVID-19 pandemic on mental health among children and adolescents. In this study, the results indicated that depressive and anxiety symptoms were common, although the ranges were wide. Other symptoms that were found to be common were irritability and anger. A systematic mixed studies review conducted by Levante, A. et al. [16] showed that the internalizing/externalizing symptoms among children increased during COVID-19, with girls exhibiting more internalizing symptoms. Nonetheless, in a systematic review conducted by Samji H. et al. [17] the authors also concluded higher anxiety and depressive symptoms among children and adolescents during COVID-19, with certain groups, such as older adolescents, more likely affected.

Despite the breadth of international research, there is a gap in large, multi-city studies in Saudi Arabia that explore the psychological impact of distance learning on children and adolescents. This research aims to bridge that gap by examining how remote education during the pandemic has affected students’ mental health and educational experiences across different demographics. More concisely, this study aims to answer the question of the psychological effect of distance learning on children and adolescents in Saudi Arabia. The findings will provide valuable insights to inform mental health advocacy and guide the future direction of education, particularly in situations where distance learning becomes necessary.

## 2. Methods

### 2.1. Ethical Considerations

The King Saud University—College of Medicine’s IRB (application number E-22-6867) granted approval for this study. Parents consented at the survey’s start, and completion was required for participation. Participants were informed of the study’s purpose and confirmed consent by clicking “yes”. Data were encrypted for confidentiality and accessed only by the research team.

### 2.2. Study Design and Participants

We employed a cross-sectional study design, distributing the e-questionnaire to parents via social media platforms, including WhatsApp. Data collection occurred between October and the end of November 2022. The sample size “n” was calculated to be 335 participants. According to the Saudi Ministry of Education’s statistics, this calculation was based on the estimated population of school-aged children in the Riyadh and Jeddah regions, which is approximately 2,000,000. The following formula was used to determine the appropriate sample size:

*x = Z*(*^c^*/_100_)^2^*r*(100 − *r*)

*n = ^N x^*/_((*N* − 1)*E*_^2^_ + *x*)_

*E =* Sqrt [^(*N* − *n*)*x*^/*_n_*_(*N* − 1)_]

This formula ensures that the sample size is sufficient to accurately represent the target population, accounting for population variability and desired precision.

Sample Size Calculator by Raosoft, Inc. 6645 NE Windermere Rd, Seattle, WA 98115, USA.

### 2.3. Inclusion and Exclusion Criteria

The study included parents of male and female children and adolescents aged 6 to 18 years who attended governmental, national, or international schools, including both public and private institutions, within the Riyadh and Jeddah regions of Saudi Arabia.

Children and adolescents with pre-existing mental illnesses, such as anxiety or depression, before the pandemic lockdown were excluded to focus on the impact of distance learning on previously healthy individuals. Additionally, children with diagnosed neurodevelopmental disorders—including Autism Spectrum Disorder (ASD), Attention Deficit Hyperactivity Disorder (ADHD), intellectual disability, and cerebral palsy—were excluded, as were those with chronic health conditions such as diabetes.

### 2.4. Tools and Procedures

The survey used for the study consisted of an online questionnaire divided into two sections:

1. 1. Sociodemographic and Background Information:

This section collected data on participants’ demographics, medical and psychiatric history, educational settings, and the level of school support provided during the pandemic.

**Figure 1:**
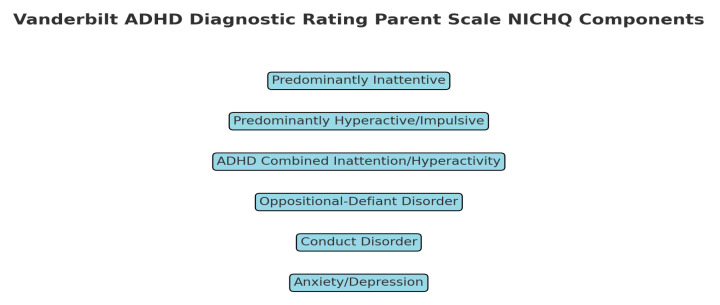
Vanderbilt ADHD Diagnostic Rating Parent Scale NICHQ Components.

2. Vanderbilt Assessment Scale:

This section employed the Vanderbilt Assessment Scale, which has been validated for use in Arabic. This scale was integrated into the survey without copyright restrictions, thanks to its accessibility through the Saudi ADHD Society’s online resources [18]. While its primary purpose is to assess the diagnosis of ADHD, it is also designed to screen for additional behavioral and emotional concerns in children 6 years and above [19]. Furthermore, it is important to note that the diagnosis of ADHD cannot be established solely through an assessment scale; hence, the reported ADHD refers to ADHD symptoms rather than a diagnosis. The e-questionnaire contains 54 items with 6 main domains (Figure 1). We confirm that we adhered to the specified criteria throughout our study, following the Scoring Instructions for the NICHQ Vanderbilt Assessment Scales for each diagnostic category:

  - Predominantly Inattentive subtype: We verified that participants scored a 2 or 3 on at least 6 out of 9 items on questions 1–9 and achieved a score of 4 or 5 on one or more of the Performance questions (questions 48–55).
  - Predominantly Hyperactive/Impulsive subtype: We ensured that participants met the requirement of scoring a 2 or 3 on at least 6 out of 9 items on questions 10–18, in addition to a score of 4 or 5 on one or more of the Performance items (questions 48–55).
  - ADHD Combined Inattention/Hyperactivity subtype: Participants were confirmed to meet the above criteria for both inattention and hyperactivity/impulsivity.
  - Oppositional-Defiant Disorder (ODD) Screen: Participants were evaluated to meet the requirement of scoring a 2 or 3 on at least 4 out of 8 items on questions 19–26 and a 4 or 5 on any Performance items (questions 48–55).
  - Conduct Disorder Screen: We confirmed that participants scored a 2 or 3 on a minimum of 3 out of 14 items on questions 27–40 and met the requirement of a 4 or 5 on the Performance items (questions 48–55).
  - Anxiety/Depression Screen: For this category, we ensured participants scored a 2 or 3 on at least 3 out of 7 items on questions 41–47 and a 4 or 5 on any Performance items (questions 48–55).

Each criterion was consistently applied based on the Scoring Instructions for the NICHQ Vanderbilt Assessment Scales to ensure accuracy and adherence to the study’s diagnostic parameters.

### 2.5. Validation and Data Collection

Parents with more than one child were allowed to complete the survey for each child. To identify multiple responses from the same household while maintaining anonymity, participants provided only the last four digits of their 10-digit family ID number. Data collected during the pilot phase were excluded from the final analysis. On average, it took parents 15 min to complete the survey. Participants self-identified by responding to invitations sent through social media platforms.

### 2.6. Statistical Analysis

Data were summarized using means ± standard deviations for continuous variables and frequencies with percentages for categorical variables. The Kolmogorov–Smirnov test was used to test the normality of the continuous variables. All statistical analyses were performed using the Statistical Package for Social Studies (SPSS) version 22 (IBM Corp., New York, NY, USA). Responses from the pilot survey were excluded from the final analysis.

Comparisons were made between children exhibiting ADHD symptoms (regardless of subtype) and those without any symptoms. The Chi-square and Fisher’s exact tests were used for categorical variables, while *t*-tests were applied for continuous variables. Homogeneity was tested by Levene’s test. Multiple logistic regression was performed to evaluate the effect of significant variables on having ADHD diagnosis as a binary response variable (Yes/No). All predictors which were significant in the univariate crosstabulation were included in the model. The regression model was controlled for age as a covariant. A *p*-value < 0.05 was considered statistically significant.

## 3. Results

The study involved 335 student participants shown in Table 1, with a mean age of 9.8 SD 3.2 years. Most families (72%) had three or more children, with 91.1% of parents married and 55% with both parents employed. Income varied, with 36.4% earning 10,000–20,000 SAR and 26.8% earning over 30,000 SAR. At the pandemic’s start, 48.7% of children were in early primary grades. Remote learning was prevalent, lasting one and a half years for 42.7% and two years for 41.2% of children, while 6.3% experienced schooling delays, primarily due to COVID-19 exposure concerns. Private schools were most common (40.9%), followed by government (36.7%) and international schools (22.4%). School changes affected 19.1% of students. Educational support came from family members in 28.7% of cases and domestic helpers in 9.3%. Almost all families (89.9%) had sufficient electronic devices for home education, with 52.2% rating distance learning as good. Chronic physical illnesses were reported in 4.5% of children, with ADHD and anxiety most frequently noted. A majority (93.7%) were not on medication, though 15% exhibited ADHD symptoms.

Table 2 presents the findings from the Vanderbilt ADHD Diagnostic Rating Parent Scale. The results show that 5.4% of the children were classified as predominantly inattentive presentation, 1.8% as predominantly hyperactive/impulsive presentation, and 3.9% as combined ADHD presentation. Additionally, 7.2% of students were screened as potentially having oppositional defiant disorder, 1.5% for conduct disorder, and 6.6% for anxiety or depression.

Table 3 compares the demographic characteristics of students with and without ADHD symptoms, showing that 50 students in the sample exhibited ADHD symptoms. There were no significant differences in the number of children in the household, the child’s age, parent’s employment status, the length of remote learning, or school delays related to the pandemic. However, significant differences were observed in parental marital status. Specifically, 25.6% of children with ADHD symptoms came from separated or divorced families, compared to only 5.4% of children without ADHD symptoms. Furthermore, a higher proportion of children with ADHD were enrolled in grades 4–6 at the beginning of the pandemic.

Table 4 highlights the academic performance and other characteristics of students with and without ADHD symptoms. No significant differences were found regarding school type, school changes due to the pandemic, or access to electronic devices at home. However, significant differences were observed in the assistance children received for distance learning. Among children with ADHD symptoms, 40% received help from family members, such as grandparents or siblings, compared to 26.8% of children without ADHD symptoms. Additionally, 18% of children with ADHD relied on domestic helpers for assistance, compared to only 7.7% of children without ADHD (*p* = 0.032). Children with ADHD also reported a more positive overall distance learning experience compared to their non-ADHD peers. However, they had a higher likelihood of being diagnosed with chronic physical illnesses during the pandemic. In terms of academic outcomes, children with ADHD symptoms exhibited higher symptom scores and lower academic performance than those without ADHD symptoms.

The multivariate logistic regression analysis results, summarized in Table 5, further explore factors associated with ADHD symptoms. The analysis found no significant link between age and ADHD symptoms. However, parental marital status was identified as a significant factor, with children from separated or divorced families being more likely to exhibit ADHD symptoms (Odds Ratio [OR] = 4.79, 95% Confidence Interval [CI]: 1.3–12.8, *p* = 0.002). Children with ADHD symptoms also reported a more positive learning experience during distance learning (OR = 5.473, 95% CI: 1.4–21.5, *p* < 0.05).

The analysis also examined whether a diagnosis of chronic physical illnesses or mental disorders during the pandemic was associated with ADHD symptoms. Although the odds ratio (OR = 2.182) was elevated, the association was not statistically significant (*p* = 0.31). No significant associations were found between ADHD symptoms and assistance from family members or domestic helpers with educational platforms. The analysis accounted for multiple children from the same household, identifying 69 children with one to three siblings, where parents completed multiple survey forms. However, the data did not reveal a significant association between having siblings from the same household and ADHD symptoms, and this factor was not further explored in the study.

## 4. Discussion

This study aimed to evaluate the behavioral and functional effects of distance learning, brought on by the COVID-19 pandemic, on children and adolescents in two major Saudi Arabian cities. Interestingly, our study found that children with ADHD reported a more positive distance learning experience compared to their non-ADHD peers.

The results of our study contrast with previous findings suggesting that students with ADHD typically struggle in remote education environments due to difficulties with attention, time management, and motivation [20,21]. However, our results align with emerging research indicating that certain children with ADHD benefit from the flexibility of remote learning [22]. The home environment may allow them to learn at their own pace, receive tailored support from parents, and avoid overstimulation common in traditional classrooms. Nevertheless, despite the reported positive experience, students with ADHD in our sample exhibited higher symptom severity and lower academic performance, consistent with other studies highlighting the academic challenges ADHD students face even in favorable environments [20].

Our study also found that children with ADHD were five times more likely to be diagnosed with chronic physical illnesses during the pandemic compared to their non-ADHD peers. This aligns with research showing a higher prevalence of chronic health conditions, such as asthma, obesity, and sleep disorders, among individuals with ADHD [23,24]. These comorbid conditions may further complicate the academic and emotional challenges experienced by children with ADHD, underscoring the need for integrated healthcare and educational support.

Furthermore, the findings revealed a significant correlation between parental marital status and ADHD symptoms, with children from separated or divorced families more than six times as likely to display ADHD symptoms compared to those with married parents controlled for their age. These results align with previous studies showing that parents of children with ADHD are more likely to experience marital discord and separation [25]. However, it is unclear whether ADHD symptoms exacerbate marital conflict or result from it. Family stability is recognized as essential in promoting healthy child behavior and mental health [26,27]. Similarly, a study conducted in Italy found that children with married parents experienced fewer traumatic effects during the COVID-19 pandemic, emphasizing the role of family dynamics during stressful times [28].

The emotional challenges for parents and educators during remote learning have been widely documented. Research from Germany, for example, reported heightened levels of stress and anxiety among parents as they took on teaching responsibilities at home, with children requiring intensive supervision to remain engaged in online classes [29]. Similarly, a systematic review by Carrión-Martínez et al. [30] found increased verbal aggression, family tension, and reduced well-being across students, parents, and teachers. The increased household stress identified in these studies aligns with our findings, suggesting that family environments significantly affect children’s ability to manage distance learning challenges.

Our study emphasizes the protective role of family support during remote education. Children who received help from siblings or relatives were less likely to exhibit ADHD symptoms than those who relied on domestic helpers. This finding aligns with prior research indicating that family involvement improves academic outcomes and reduces behavioral challenges [31]. Shahali et al. [32] reported that children with learning difficulties performed better academically when provided with structured support and consistent family involvement during the pandemic.

Interestingly, our study found that children with ADHD reported a more positive distance learning experience compared to their non-ADHD peers. This contrasts with previous findings suggesting that students with ADHD typically struggle in remote education environments due to difficulties with attention, time management, and motivation [20,21]. However, our results align with emerging research indicating that certain children with ADHD benefit from the flexibility of remote learning [22]. The home environment may allow them to learn at their own pace, receive tailored support from parents, and avoid overstimulation common in traditional classrooms. Nevertheless, despite the reported positive experience, students with ADHD in our sample exhibited higher symptom severity and lower academic performance, consistent with other studies highlighting the academic challenges ADHD students face even in favorable environments [20]. Our findings emphasize the importance of family involvement and structured support during stressful events like the pandemic. Studies from the U.S. and Europe show that family cohesion and proactive engagement are critical in mitigating adverse outcomes during distance learning [9,33]. Moreover, children who receive consistent support from siblings or parents are more likely to develop resilience and emotional stability [34]. Conversely, reliance on external caregivers, such as domestic helpers, may introduce inconsistencies in children’s routines, contributing to behavioral issues. This finding underscores the need for schools and mental health professionals to engage families actively in intervention strategies, especially when managing children with ADHD.

The strength of this study lies in its scope as the first large-scale, multi-city investigation in Saudi Arabia to examine the impact of distance learning on children’s mental health. By using the validated Arabic Vanderbilt ADHD Diagnostic Rating Scale, we ensure reliable measurement of behavioral and academic outcomes, contributing to a relatively under-researched area. Additionally, the study highlights the importance of family engagement in helping children manage stress during challenging times.

The findings offer valuable insights for mental health professionals and educators. These findings suggest that flexible learning models and family-centered interventions can improve outcomes for children with behavioral challenges. As the pandemic reshapes educational practices, mental health professionals and policymakers must collaborate to create adaptive learning environments that meet the needs of all students, particularly those with ADHD and other behavioral disorders.

In addition, it emphasizes the need for family-centered interventions and flexible learning environments to support children with behavioral challenges. Further research should explore additional chronic health conditions and investigate how remote learning outcomes vary across different socioeconomic and cultural contexts.

### 4.1. Practical Implication

This study adds to a growing body of research showing that children with ADHD and other behavioral challenges face unique difficulties during remote learning. In particular, the positive distance learning experience reported by ADHD students in our study suggests that educational policies should explore flexible learning models that accommodate children’s individual needs. For instance, remote learning may offer benefits such as reduced distractions and a more structured environment, which can help ADHD students focus better than in traditional classroom settings. However, it also highlights the importance of balancing flexibility with the need for social interaction and hands-on learning. Future research could further investigate the specific features of remote learning environments—such as flexible schedules, personalized attention, and the use of technology to support individualized learning—that may benefit children with ADHD. Additionally, examining the role of parental involvement, peer interaction, and the design of online curricula could provide further insights into how to optimize learning experiences for these students. Understanding these nuances will be critical for developing more inclusive educational strategies and interventions to support children with ADHD in both remote and in-person learning settings.

### 4.2. Limitations and Prospective Directions

This study contributes to the limited research on the effects of the COVID-19 pandemic on the well-being of school-aged children, but several limitations should be considered. The reliance on an online survey may have excluded families with limited technology access, potentially skewing the sample toward higher-income households. Although the survey was distributed through school platforms, snowball sampling could be attributed to technology gaps or parents being overwhelmed with caregiving responsibilities, limiting the statistical power and scope for follow-up.

While the diverse responses from families in major Saudi cities enhance the study’s generalizability, relying solely on parental reports introduces potential bias. This could lead to the Horn effect, where parents of children with severe symptoms might overestimate the negative impact. The absence of teacher input or self-reports from older children further limits the study, especially since no validated self-report tools were available in the local context.

The use of predefined categories, with only a sixth “other” option, may have discouraged detailed reporting of less common conditions. To address these gaps, future research should broaden its scope to include multiple disabilities and allow for comparisons across different age groups.

Given the possibility of selection bias, the findings should be interpreted cautiously. Future studies should seek to integrate parental and teacher perspectives alongside child self-reports to gain a more holistic understanding of the pandemic’s impact. A more comprehensive approach would provide insights into the interaction between mental health, family dynamics, and educational outcomes across diverse socioeconomic contexts.

## Figures and Tables

**Table 1:** Characteristics of the students and their parents (“n” = 334).

Variable	Group	Number	%	Variable	Group	Number	%
The number of children in the home “n” = 280	1	54	19.3	What is the reason for the delay? “n” = 21	financial conditions	1	4.8
	2	78	27.9		social conditions	1	4.8
	3	71	25.4		Fear of exposure to the Coronavirus while having a health condition	4	19
	4	48	17.1		Fear of exposure to the Coronavirus and the child’s health condition is very good	8	38.1
	5	14	5		Another reason	7	33.3
	>5	15	5.4	School type	government	123	36.7
Parents’ marital status “n” = 280	married	255	91.1		private	137	40.9
	separated or divorced	23	8.2		International	75	22.4
	One of the parents is deceased	2	0.7	Has the school changed due to the pandemic?	Yes	64	19.1
The age of the child at the beginning of the pandemic (Mean, SD)		9.8	3.2		No	270	80.6
Parent’s employment status Mother and father are working “n” = 280 Only mother works Only father works Mother and father do not work		154	55	Were others, such as grandfather, grandmother, or one of the siblings, used to help the child attend the educational platform?	Yes	96	28.7
		16	5.7		No	239	71.3
		94	33.6	2 Was a maid relied upon to help the child attend the educational platform?	Yes	31	9.3
		16	5.7		No	304	90.7
The total monthly income of the family “n” = 280	<10,000 SR	31	11.1	The presence of sufficient electronic devices for education at home	Yes	301	89.9
	10,000–20,000 SR	102	36.4		No	34	10.1
	20,000–30,000 SR	72	25.7	Assess the overall learning experience of distance learning for your respective child.	very bad	40	11.9
	>30,000 SR	75	26.8		bad	55	16.4
The child’s classroom at the beginning of the pandemic—March 2020 “n” = 335	1–3 Primary	163	48.7		acceptable	65	19.4
	4–6 Primary	92	27.5		good	78	23.3
	intermediate	57	17		very good	55	16.4
	secondary	23	6.9		excellent	42	12.5
The child’s grade level when attendance or semi-attendance learning began again “n” = 335	1–3 Primary	120	35.8	Did your child suffer from chronic physical illnesses before the pandemic?	No	335	100
	4–6 Primary	111	33.1	Has your child been diagnosed with chronic physical illnesses during the pandemic?	Yes	15	4.5
	intermediate	62	18.5		No	320	95.5
	secondary	42	12.5	What is the diagnosis?	ADHD	5	38
The number of academic years the child studied remotely from March 2020 to March 2022 “n” = 335	half a year	21	6.3		Anxiety	4	31
	one year	33	9.9		get distracted	2	15
	A year and a half	143	42.7		psychosis	1	8
	two years	138	41.2		A combination of distractibility, hyperactivity, learning difficulties, and delay in social communication skills	1	8
Is your child late for school because of Corona? “n” = 335	Yes	21	6.3	Was this assessment done at a time when the child was on meds?	He takes medication	10	3
	No	314	93.7		He does not take medication	314	93.7
If the answer is 1, how many years later? “n” = 21	half a year	11	52.4		I’m not sure	11	3.3
	one year	5	23.8				
	more than one year	5	23.8				

**Table 2:** Vanderbilt ADHD Diagnostic Rating Parent Scale NICHQ.

	Number	%
Predominantly Inattentive subtype	18	5.4
Predominantly Hyperactive/Impulsive subtype	6	1.8
ADHD Combined Inattention/Hyperactivity	13	3.9
Oppositional-Defiant Disorder Screen	24	7.2
Conduct Disorder Screen	5	1.5
Anxiety/Depression Screen	22	6.6

**Table 3:** Comparison between students with any ADHD symptom and those without and other demographic characteristics.

		Are There Any ADHD Symptoms				*p* Value *
		No “n” = 284		Yes “n” = 50		
		Number	%	Number	%	
The number of children in the home	1	45	18.8	9	23.1	0.81
	2	68	28.3	9	23.1	
	3	60	25	11	28.2	
	4	40	16.7	8	20.5	
	5	13	5.4	1	2.6	
	>5	14	5.8	1	2.6	
Age	mean SD	9.8	3.2	9.1	3.1	0.353
Parents’ marital status	married	225	93.8	29	74.4	0.001 *
	separated or divorced	13	5.4	10	25.6	
	One of the parents is deceased	2	0.8	0	0	
What is the reason for the delay?	financial conditions	1	5.6	0	0	0.701
	social conditions	1	5.6	0	0	
	Fear of exposure to the Coronavirus while having a health condition that makes it vulnerable to complications	4	22.2	0	0	
	Fear of exposure to the Coronavirus and the child’s health condition is very good	7	38.9	1	33.3	
	Another reason	5	27.8	2	66.7	

* *p* value was estimated by chi-square or student test.

**Table 4:** ADHD combines inattention/Hyperactivity of the student by their characteristics.

		Are There Any ADHD Symptoms				*p* Value *
		No “n” = 284		Yes “n” = 50		
		Number	%	Number	%	
School type	government	102	35.9	21	42	0.7
	private	117	41.2	19	38	
	International	65	22.9	10	20	
Has the school changed due to the pandemic?	Yes	52	18.4	12	24	0.337
	No	231	81.6	38	76	
Were others, such as grandfather, grandmother, or one of the siblings2, used to help the child attend the educational platform?	Yes	76	26.8	20	40	0.06
	No	208	73.2	30	60	
Was a maid relied upon to help the child attend the educational platform?	Yes	22	7.7	9	18	0.032 *
	No	262	92.3	41	82	
The presence of sufficient electronic devices for education at home	Yes	254	89.4	46	92	0.8
	No	30	10.6	4	8	
Assess the overall learning experience of distance learning for your respective child	very bad	38	13.4	2	4	0.002 *
	bad	51	18	4	8	
	acceptable	54	19	10	20	
	good	69	24.3	9	18	
	very good	44	15.5	11	22	
	excellent	28	9.9	14	28	
Has your child been diagnosed with chronic physical illnesses during the pandemic?	Yes	8	2.8	7	14	0.003 *
	No	276	97.2	43	86	
Total symptom	mean SD	2.6	3.2	8.8	4.7	0.001 *
Average performance	mean SD	0.131	0.211	0.523	0.291	0.001 *

* *p* value was estimated by chi-square or student test.

**Table 5:** Multiple logistic regression for the associated factors with ADHD Symptoms.

		Odds Ratio	95% C.I. for OR		*p* Value
			Lower	Upper	
Age		1.008	0.895	1.137	0.89
Parents’ marital status	Married **	1			
	separated or divorced	4.79	1.787	12.836	0.002 *
Were others, such as grandfather, grandmother, or one of the siblings, used to help the child attend the educational platform?	No **	1			
	Yes	1.556	0.708	3.421	0.271
Was a maid relied upon to help the child attend the educational platform?	No **	1			
	Yes	1.032	0.322	3.309	0.957
Has your child been diagnosed with chronic physical illnesses such as diabetes, mental disorders such as anxiety or depression, developmental disorders such as autism or ADHD, or mental retardation during the pandemic?	No **	1			
	Yes	2.182	0.479	9.937	0.313
Assess the overall learning experience of distance learning for your respective child	very bad/bad **	1			
	acceptable	5.473	1.392	21.514	0.015 *
	good	3.211	0.781	13.206	0.106
	very good/excellent	6.902	1.831	26.012	0.004 *

* *p* value < 0.05; ** Reference group.

## Data Availability

The data supporting this study’s findings are available upon reasonable request from the corresponding author.

## Abbreviations

ADHD	Attention Deficit Hyperactivity Disorder
GAD 7	Generalized Anxiety Disorder—7 Items
COVID-19	CoronaVirus Disease of 2019
DASS 21	The Depression: Anxiety, and Stress Scale —21 c
ID	Identifying Information
